# Ipilimumab: A First-in-Class T-Cell Potentiator for Metastatic Melanoma

**DOI:** 10.1155/2013/423829

**Published:** 2013-04-21

**Authors:** Bartosz Chmielowski

**Affiliations:** Division of Hematology/Oncology, Department of Medicine, University of California, Los Angeles, Los Angeles, CA 90095, USA

## Abstract

Ipilimumab, a fully human anti-cytotoxic T-lymphocyte antigen-4 monoclonal antibody that potentiates antitumor T-cell responses, has demonstrated improved survival in previously treated and treatment-naïve patients with unresectable stage III/IV melanoma. Survival benefit has also been shown in diverse patient populations, including those with brain metastases. In 2011, ipilimumab (3 mg/kg every 3 weeks for 4 doses) was approved by the Food and Drug Administration for unresectable or metastatic melanoma. Ipilimumab can induce novel response patterns for which immune-related response criteria have been proposed. irAEs are common but are usually low grade; higher grades can be severe and life-threatening. irAEs are usually manageable using established guidelines emphasizing vigilance and prompt intervention. This agent provides an additional therapeutic option in metastatic melanoma, and guidelines for management of adverse events facilitate clinical implementation of this new agent.

## 1. Introduction

The incidence of melanoma has more than tripled in the Caucasian population during the last 20 years, and melanoma currently is the sixth most common cancer in the United States [1]. Recent estimates indicate that approximately 70,230 Americans (40,010 men and 30,220 women) developed invasive cutaneous melanoma in 2011, and 53,360 cases of melanoma *in situ* will be reported [1]. Although melanoma accounts for only 4% of all skin cancers, it is responsible for approximately 80% of all skin cancer deaths [2] with an estimated 8790 deaths from melanoma in 2011 [3]. For the majority of patients, the diagnosis of melanoma occurs at an early stage; 84% are diagnosed with localized disease. In contrast, for the small percentage of patients with a first diagnosis of unresectable stage III or stage IV or for those who recur with advanced disease, the associated clinical burden is significant and the prognosis is poor. For the 8% of patients diagnosed with stage III disease, 5-year relative survival is 62% [4]. For the 4% of patients diagnosed with unresectable stage III or IV (advanced) disease, historical benchmark data from a recent meta-analysis estimates a 25% 1-year survival [5], falling to approximately only 15% by 5 years [4, 5]. 

In addition to poor survival, patients diagnosed with advanced melanoma have limited treatment options: dacarbazine remains the only chemotherapy approved for use in the United States [6]. However, dacarbazine is associated with modest response rates (7–12%) and has never been tested in a randomized clinical trial setting for the purposes of evaluating improvement in overall survival [7]. The low response rates achieved with dacarbazine monotherapy in melanoma have since been confirmed in two recent phase III trials employing dacarbazine as a control therapy; the trials yielded overall response rates of approximately 5% [8] and 10% [9]. 

The first immunotherapy to be approved by the Food and Drug Administration (FDA) for treatment of advanced melanoma was interleukin-2 (IL-2) but, like dacarbazine, response rates are low. High-dose bolus IL-2 achieved objective responses in only 5–27% of patients and complete response in 0–4% in three randomized trials [6]. Furthermore, clinical utility of IL-2 has been limited by significant dose-dependent toxicity, which though reversible, is often severe [7, 10]. Until recently, no therapeutic regimen has been shown to prolong survival in randomized, phase III trials, so enrollment in a clinical trial has been the preferred management option [6, 11].

In 2011, two new therapies were approved by the FDA and are now available for use in patients with advanced melanoma. Ipilimumab is a fully human monoclonal antibody that potentiates antitumor T-cell responses and has demonstrated improved overall survival in two phase III studies in previously treated and treatment-naïve patients with unresectable stage III or IV melanoma [9, 12]. Ipilimumab is FDA approved for the treatment of patients with unresectable or metastatic melanoma. Although response rates were moderate (5.7–10.9% used ipilimumab as a single agent in previously treated patients, and 15.2% used it in combination with dacarbazine in treatment-naïve patients), some patients experienced a durable control of the disease. Median duration of response was 11.5 months in previously treated patients and 19.3 months in treatment-naïve patients [9, 12]. The second therapy approved is vemurafenib, a BRAF inhibitor, which has shown improved 6-month overall survival (84% versus 64%; *P* < 0.001) and significantly higher response rates (48% versus 5%; *P* < 0.001) compared with dacarbazine in a phase III study (BRIM3) of treatment-naïve patients with metastatic melanoma [8]. A subsequent survival update from a single-arm phase II study of vemurafenib (BRIM2) reported a median overall survival (OS) of 15.9 months and a 1-year survival rate of 58% after a median follow-up of 12.9 months [13]. Duration of responses to vemurafenib may be limited by the development of resistance [14]. In addition, only patients whose tumors harbor the V600 mutation can benefit from vemurafenib, hence its approval only for the treatment of patients with unresectable or metastatic melanoma with BRAF V600E mutation [8, 13]. This review focuses on the efficacy and safety of ipilimumab and highlights management of treatment-related adverse events (AEs).

## 2. Ipilimumab as an Immunotherapeutic Approach

**Figure 1:**
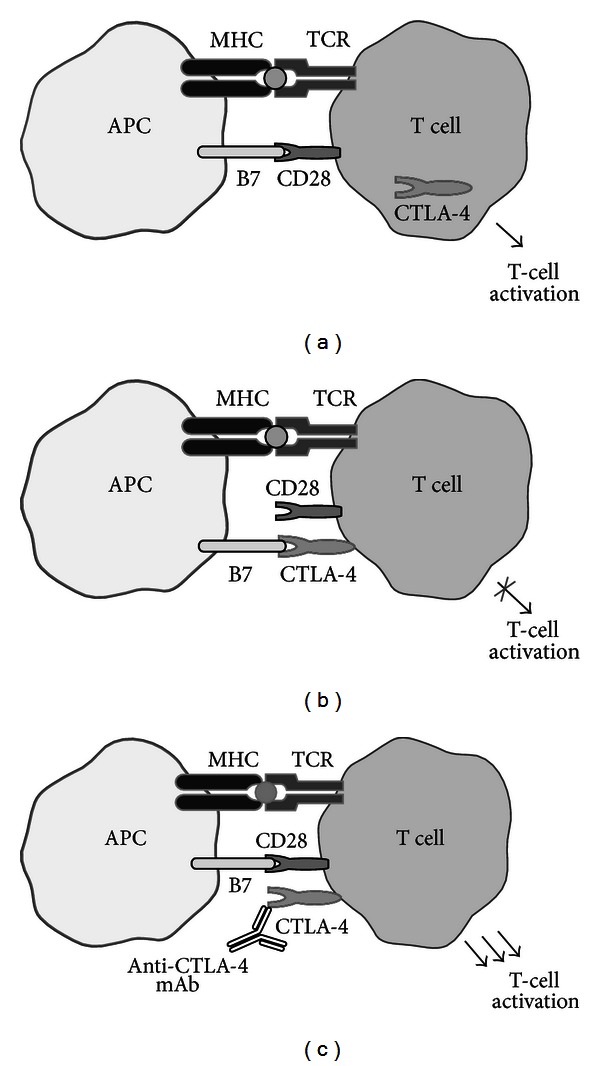
Role of CTLA-4 in T-cell responses and the impact of CTLA-4 blockade with ipilimumab. (a) Two signals are required for activation of T-cells. (b) Upon activation, CTLA-4 is upregulated, and once bound to the costimulatory molecule it prevents further immune activation. (c) Ipilimumab binds CTLA-4 thus augmenting T-cell response.

Increased understanding of how the immune system interacts with a tumor and its microenvironment has led to the identification of novel targets for evaluation as potential new immunotherapies [15]. T-cell-mediated antitumor therapies, such as IL-2, have played an important role in progressing immunotherapeutic approaches to the treatment of advanced melanoma [16]. T-cell activation is a highly regulated process which requires 2 signals [15–18]. Tumor-associated antigens attached to the major histocompatibility complex I or II on specialized antigen-presenting cells (APCs) bind with T-cell receptors (Figures 1(a) and 1(b)). T-cell activation is initiated when B7 molecules on the APC surface bind with CD28 receptors on the T-cell surface. Activated T-cells subsequently proliferate and target the tumor. Shortly after T-cell activation, cytotoxic T-lymphocyte antigen-4 (CTLA-4) is upregulated to competitively inhibit the binding of B7 to CD28 and thus stop T-cell activation and proliferation. CTLA-4 knockout mice have a massive, CD28-dependent expansion of autoreactive T-cells and die within 3 to 4 weeks due to extensive lymphoproliferation and lymphadenopathy, evidence of the significant role CTLA-4 plays in inhibiting the activation and proliferation of T-cells. Ipilimumab is a fully human anti-CTLA-4 antibody that blocks CTLA-4 and therefore augments antitumor T-cell responses (Figure 1(c)) [15, 19]. 

Early studies in mice and primates, and later in humans, demonstrated that ipilimumab competitively binds to CTLA-4 more efficiently than B7 while preserving CD28 signaling [15, 20, 21]. The efficacy and safety of ipilimumab at various doses was demonstrated in a clinical trial program that has treated over 3000 patients with advanced melanoma in randomized phase II trials and provided strong support for the concept of blockade of CTLA-4 as an immunotherapeutic approach [22–25]. More recently, ipilimumab has demonstrated efficacy and safety in two randomized, multicenter phase III trials. In the MDX010-20 study, previously treated patients with unresectable stage III or IV melanoma were randomized to receive ipilimumab alone (*n* = 136), ipilimumab plus the experimental vaccine glycoprotein 100 (gp100; *n* = 403), or gp100 alone (*n* = 136). With or without the gp100 vaccine, ipilimumab decreased the risk of death by 32–34% and significantly increased median overall survival (*P* < 0.001) compared with gp100 alone. At a median follow-up of 17.2–27.8 months, median overall survival for ipilimumab alone was 10.1 months, ipilimumab plus gp100 was 10.0 months, and gp100 alone was 6.4 months [12]. The MDX010-20 study served as the registrational trial for FDA approval. The efficacy and safety profile of ipilimumab was further supported in the phase III CA184-024 study. In this trial, treatment-naïve patients with unresectable stage III or IV melanoma received either dacarbazine plus placebo or a combination of ipilimumab and dacarbazine; addition of ipilimumab to dacarbazine decreased the risk of death by 28% compared with dacarbazine alone. Median overall survival for ipilimumab-dacarbazine was 11.2 months versus 9.1 months (*P* < 0.001) in the dacarbazine plus placebo group [9]. Of note, the CA184-024 study used a higher experimental dose of ipilimumab (10 mg/kg) than the 3 mg/kg approved dose used in the registrational MDX010-20 study and had a maintenance phase for eligible patients. Data indicate that ipilimumab can achieve long-term survival for some patients, to date, up to 2 to 3 years in phase III studies and up to 4 years in phase II studies [26, 27].

## 3. Efficacy in Patient Subpopulations

Ipilimumab demonstrated improved overall survival in both previously treated and treatment-naïve patients. In both phase III trials, the survival benefit demonstrated with ipilimumab was apparent across patient subgroups and regardless of negative prognostic factors such as performance status, age or gender, baseline serum lactate dehydrogenase (LDH) level, or metastatic disease substage [9, 12, 28]. There is also no impact of BRAF mutational status on ipilimumab activity [28]. Ipilimumab has been FDA approved for all patients with unresectable or metastatic melanoma, and there are no restrictions regarding its use in specific patient populations [29]. 

The registrational MDX010-20 study allowed the inclusion of patients with stable brain metastasis. A total of 77 patients with a history of brain metastases received treatment: 42 in the ipilimumab-gp100 group, 15 in the ipilimumab-alone group, and 20 in the gp100-alone group. Subgroup analysis demonstrated that ipilimumab was active in these patients and they had a similar immune-related AE (irAE) profile following ipilimumab treatment as patients with no history of brain metastases [30]. These findings have been confirmed in subanalyses of patients with advanced melanoma and stable asymptomatic brain metastases (*n* = 165) who entered the ipilimumab expanded access program. These patients demonstrated durable responses, with no increase in the occurrence of central nervous system- (CNS) related toxicities or unique toxicities observed [31]. Further confirmation has been reported in results from a prospective phase II study in patients with melanoma and symptomatic brain metastases. OS for patients with asymptomatic metastases was 31% at 1 year and 26% at 2 years, whereas symptomatic patients had rates of 19% at 1 year and 10% at 2 years. As in previous reports, the safety profile revealed no new signals and there was no increase in CNS-related events [32]. 

The efficacy and safety profile of ipilimumab appears to be similar among elderly patients (65 years and over) and younger patients (<65 years), and no overall differences have been reported. However, the safety and efficacy of ipilimumab has not been established in pediatric patients, and no formal studies of ipilimumab in patients with renal or hepatic impairment have been conducted [29]. It is also not known whether ipilimumab is secreted in human milk. Because of the potential for serious adverse reactions in nursing infants from ipilimumab, a decision should be made whether to discontinue nursing or to discontinue ipilimumab, taking into account the potential clinical benefit of ipilimumab to the mother [29].

## 4. Pharmacokinetics of Ipilimumab

The pharmacokinetics of ipilimumab have been studied in 499 patients with unresectable or metastatic melanoma who received various doses (0.3, 3, or 10 mg/kg) administered once every 3 weeks for a total of 4 doses [29]. Peak concentration (*C*_max⁡_), trough concentration (*C*_min⁡_), and the area under curve (AUC) of ipilimumab were found to be dose proportional within the dose range examined. Based on repeated dosing of ipilimumab administered every 3 weeks, ipilimumab clearance was found to be time invariant, and minimal systemic accumulation was observed as evident by an accumulation index of 1.5-fold or less. Ipilimumab steady-state concentration was reached by the third dose. Population pharmacokinetic analyses demonstrated that following systemic ipilimumab treatment, mean terminal half-life was 14.7 days (30.1% coefficient of variation), clearance was 15.3 mL/h (38.5% coefficient of variation), and volume of distribution at steady state was 7.21 L (10.5% coefficient of variation). Mean ipilimumab *C*_min⁡_ achieved at steady state with the 3 mg/kg regimen was 21.8 mcg/mL (±11.2 standard deviation). 

Cross-study analyses were performed on data from patients with a variety of conditions, including 420 patients with melanoma who received single or multiple infusions of ipilimumab at doses of 0.3, 3, or 10 mg/kg. The effects of various covariates on ipilimumab pharmacokinetics were assessed in population pharmacokinetic analyses. Ipilimumab clearance increased with increasing body weight; however, no dose adjustment is required for body weight after administration on a mg/kg basis. Age (range 26–86 years), gender, concomitant use of budesonide, performance status, human leukocyte antigen (HLA)-A2*0201 status, positive anti-ipilimumab antibody status, prior use of systemic anticancer therapy, or baseline LDH levels had no clinically meaningful effect on the clearance of ipilimumab. Due to insufficient numbers of patients in non-Caucasian ethnic groups, the effects of race were not examined. With regard to renal impairment, creatinine clearance at baseline did not have a clinically important effect on ipilimumab pharmacokinetics in patients with calculated creatinine clearance values of 29 mL/min or greater. Furthermore, baseline aspartate aminotransferase, total bilirubin, and alanine aminotransferase levels did not have a clinically important effect on ipilimumab pharmacokinetics in patients with various degrees of hepatic impairment. Ipilimumab (induction) is administered at 3 mg/kg as a 90-minute intravenous infusion every 3 weeks for a total of 4 doses [29].

## 5. Ipilimumab-Associated Patterns of Response

**Figure 2:**
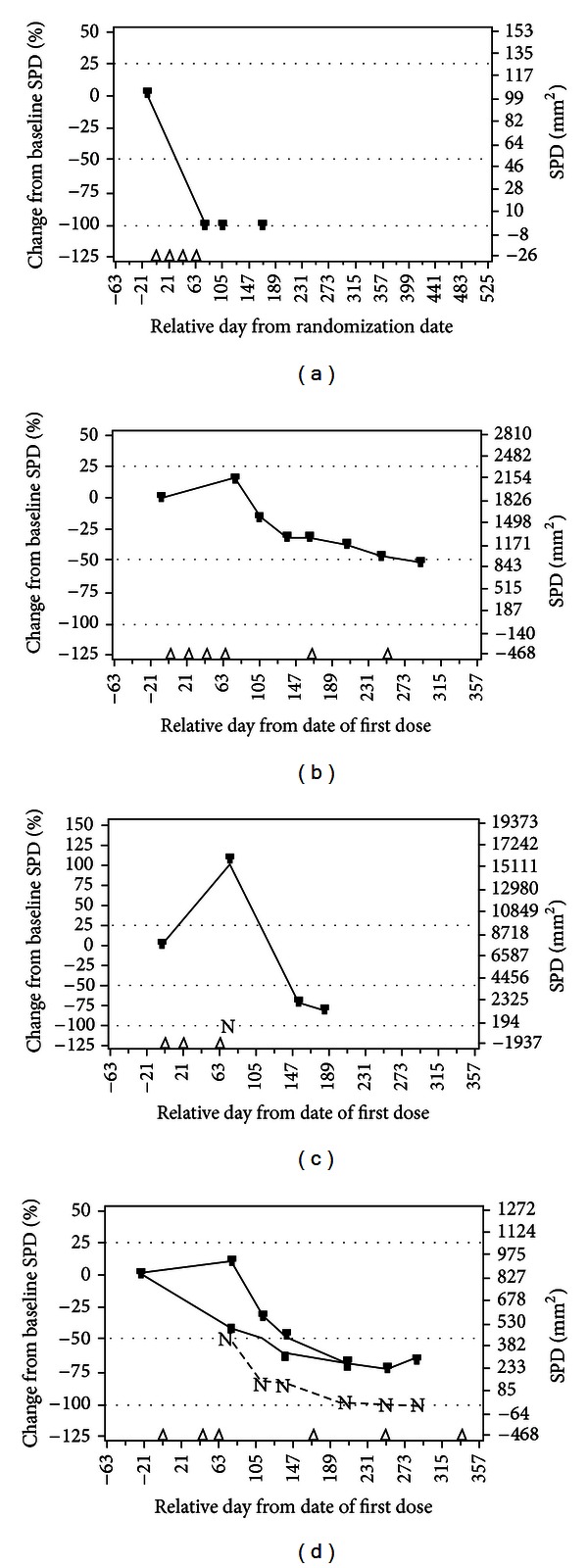
Patterns of response with ipilimumab therapy [28]. There have been 4 response patterns observed in advanced melanoma patients treated with ipilimumab at 10 mg/kg in phase II studies, and all have been associated with favorable patient outcomes. They are (a) response in baseline lesions; (b) stable disease; (c) response after initial increase in total tumor volume; and (d) reduction in total tumor burden after the appearance of new lesions. Reprinted from [28] with permission from AACR. N, tumor burden of new lesions ((c) and (d)). (d) top line, total tumor burden; middle line, tumor burden of baseline lesions; bottom line, tumor burden of new lesions. Triangles, ipilimumab dosing time points; dashed lines, thresholds for response or PD/irPD. irPD: immune-related progressive disease; PD: progressive disease; SPD: sum of the product of perpendicular diameters.

The types of clinical responses observed across studies throughout the clinical development of ipilimumab are consistent with documented patterns associated with other immunotherapeutic agents [9, 12, 28, 33]. Some, but not all, response patterns produced by ipilimumab therapy can differ from the conventional responses observed with cytotoxic agents [28]. In common with cytotoxic therapy, clinical responses to ipilimumab may include rapid decline of baseline lesions and no evidence of new lesions following treatment; stable disease, which in some cases may be followed by slow and steady decline of tumor burden [28]; and though unusual, durable, stable disease lasting months or even years has been observed [23, 26]. Unlike conventional cytotoxic therapy, ipilimumab can induce two additional novel response patterns which can appear to be a “mixed response” (Figure 2) [28]. The first of these is response after an initial increase in tumor burden, which may be associated with T-cell infiltration giving the appearance of progressive disease. The second is a reduction in total tumor burden during or after the appearance of new lesions, possibly due to the unique mechanism of action of ipilimumab, as the activated immune system may take some time to mount an effective response [28]. Although standard response criteria cannot capture all of the responses produced by novel immunotherapy, both the traditional and new response patterns are associated with favorable survival [28]. New immune-related response criteria for the evaluation of immune therapy have been proposed [28], and it has been suggested that these should be applied to patients undergoing therapy with ipilimumab for advanced melanoma [34]. From a clinical perspective, these different patterns of response make clinical decisions on discontinuation of ipilimumab challenging. Even the proposed immune-related response criteria are not able to capture the full benefit of ipilimumab. When these criteria were applied to 227 patients treated with ipilimumab at 10 mg/kg in the single-arm study CA184-008, only additional 5 patients (2.2%) were identified as responders, although they met the World Health Organization criteria for progressive disease. As previously discussed, the phase III CA184-024 study showed 28% reduction in the risk of death and 24% reduction in progression of the disease (measured by hazard ratio), but the difference in response was only 4.9% (15.2% versus 10.3%), and there was marginal improvement in the number of patients with stable disease (18% versus 19.8%). It suggests that about 10% of patients benefit from ipilimumab by reduction in the speed of tumor growth. Clinicians may find it very difficult to assess that type of benefit when they make decisions on introduction of a new therapy, and therefore it is generally believed that if patients do not have massive growth of the tumor and if their performance status is stable or improved, they could be observed for a possible delayed response; patients whose condition deteriorates should be started on another therapy. 

## 6. Safety Events Associated with Ipilimumab

The most common safety events associated with ipilimumab are immune related and most likely reflective of ipilimumab's mechanism of action [33–35]. A recent pooled analysis of 14 phase I to III studies, including 1498 patients using various doses of ipilimumab (0.1–20 mg/kg), found that 64.2% of patients enrolled in ipilimumab clinical trials experienced an irAE of any grade. The majority of irAEs were low grade (1 or 2), though severe irAEs (grade 3–5) occurred in 18.4% of patients and were fatal in 0.6% of patients. irAEs of the gastrointestinal (GI) tract and skin were most common; hepatic, endocrine, and neurologic events were less frequent [36]. Although irAEs have been reported in patients many weeks or even months after the last dose of ipilimumab, they generally have rapid onset and are typically observed within 12 weeks of initiation of therapy [37, 38]. At both the approved 3 mg/kg and investigational 10 mg/kg dose, time to onset (5-6 weeks) and time to resolution (4–8 weeks) of events are similar [37]. However, rates can vary widely depending on the organ system involved [38]. The earliest to occur are irAEs involving the skin, sometimes after only 1 or 2 doses of ipilimumab. In contrast, endocrine events occur for a median of 9-10 weeks after initiation of ipilimumab therapy. This variation is also seen in time to resolution: GI, liver, and skin irAEs usually resolve within a few weeks; endocrine events take around 20 weeks to resolve and in some cases are irreversible [38].

Given the incidence of irAEs, a standard set of management guidelines were developed throughout the course of clinical development of ipilimumab [39]. These guidelines stress vigilance and the use of corticosteroids when appropriate [40, 41]. A review of data from three phase II studies of ipilimumab used at 10 mg/kg suggested that steroids do not influence the efficacy of ipilimumab, but the data must be interpreted with caution, since it was a retrospective analysis and patients were not randomized [40, 41]. It is well established, however, that steroids can reverse irAEs, and the mechanism of these reactions is identical to the mechanism of the antitumor activity of ipilimumab [12]. It should be noted that prior to FDA approval being awarded, an additional retrospective safety analysis of MDX010-20 study subjects was required in order to exclude those events which were prospectively defined and appeared to be immune related but could later be determined to have a non inflammatory etiology. Within the US label, results of this analysis are presented as “immune-mediated adverse reactions” and generally describe similar kinds of events as irAEs, though applying a different methodology to the safety data [29]. Regardless of causality, all inflammatory events should be managed the same way. 

## 7. Management of irAEs

Throughout the clinical development program for ipilimumab, evaluation of therapy safety and especially the development of guidance on optimal management of irAEs have been part of the development strategy. As a result, and in collaboration with the FDA, a risk evaluation and mitigation strategy (REMS) has been developed with the aim of ensuring that the benefits of ipilimumab therapy are evaluated against the risks of severe and potentially fatal immune-mediated adverse reactions. REMS includes a communication plan to disseminate safety issues with AEs and guidelines for their management, and it reinforces the importance of early detection and prompt reporting to reduce serious and sometimes fatal events [39]. At baseline and at each follow-up visit, patients should be assessed for signs and symptoms of irAEs, as most low-grade (grade 1-2) events can be managed symptomatically [15, 36]. REMS includes guidance on management of GI, skin, liver, endocrine, and other irAEs (Table 1).

It has been attempted to use budesonide prophylactically for GI events. Oral budesonide is a controlled-release formulation that delivers the drug locally in the terminal ileum and ascending colon. When absorbed, it undergoes extensive first-pass metabolism that lowers its systemic bioavailability. It should be noted that with respect to ipilimumab therapy, prophylactic budesonide use is not effective in reducing the rate of grade ≥2 diarrhea; however, it can have a therapeutic effect in patients with milder cases of diarrhea [22, 42]. Furthermore, use of opioids to manage abdominal pain may mask signs of bowel perforation. In terms of liver-associated irAEs, it should be noted that elevations of liver function tests may occur in the absence of clinical symptoms. Mycophenolate treatment has been administered in patients who have persistent severe hepatitis despite high-dose corticosteroids [29]. Endocrinopathies are probably the most difficult irAE to diagnose due to the nonspecific nature of many of the signs and symptoms (Table 1). Blood work should be done, especially evaluating the pituitary gland as adrenal crisis can occur, though it is rare. Hypopituitarism and hypo- or hyperthyroidism has also been observed in studies, and long-term hormone replacement therapy may be necessary in some patients [29]. Other types of events that have been reported include neurologic and ocular events. Though irAEs can be severe in some patients, overall they are treatable, and most resolve in a reasonable amount of time if identified early and appropriate treatment is administered. The guidelines recommend that upon improvement to grade 1 or less for all irAEs, corticosteroid taper should be initiated and continue to be tapered over at least 1 month [39].

## 8. Conclusions 

Ipilimumab is the first-in-class anti-CTLA-4 therapy to be approved by the FDA for treatment of metastatic melanoma and has demonstrated efficacy in these patients. The approved regimen with ipilimumab is a 90-minute intravenous infusion at 3 mg/kg every 3 weeks for 4 doses. Although the majority of patients experience an irAE, these are usually low grade and manageable. Low-grade AEs are typically managed symptomatically, though higher-grade AEs can be severe and life-threatening. irAEs are manageable using established guidelines which emphasize vigilance and prompt intervention with steroids when appropriate. Studies demonstrate that time to onset and resolution of irAEs were relatively consistent at both the approved 3 mg/kg dose and the investigational 10 mg/kg dose but show variation according to the organs system involved. Ipilimumab is currently approved as a monotherapy, but ongoing research of combinations with other anticancer agents, both immunotherapies and chemotherapies, may ultimately change the melanoma treatment landscape by establishing additional effective therapeutic approaches without significantly impacting safety.

## Figures and Tables

**Table 1:** Guidelines for recommended management of irAEs.

Site	Signs and symptoms	Management
GI	Assess patients for changes in bowel habits and for the following signs and symptoms: diarrhea, abdominal pain, blood or mucus in stool with or without fever, peritoneal signs consistent with bowel perforation, and ileus.	Low-grade events: symptomatic management (dietary modifications and loperamide). High-grade events: corticosteroid therapy may be required. >7 stools/day over baseline, signs consistent with perforation, or patients with a fever: administer 1-2 mg/kg prednisone or equivalent and then move forward with ensuring differential diagnosis. Withhold ipilimumab for moderate reactions until improvement to mild severity or complete resolution; for severe reactions, discontinue ipilimumab.

Skin	Evaluate patients for signs and symptoms of pruritus, vitiligo, or maculopapular rash.	Mild to moderate: symptomatic management. Topical moisturizers and oatmeal baths may help relieve mild cases. Moderate to severe: topical and/or systemic corticosteroids may be required. Withhold ipilimumab dosing in patients with moderate to severe signs and symptoms. Permanently discontinue ipilimumab in patients with Stevens-Johnson syndrome, toxic epidermal necrolysis, or rash complicated by full thickness dermal ulceration or necrotic, bullous, or hemorrhagic manifestations.

Liver	Run liver function tests before each infusion or more frequently if possible. Monitor patients for any signs of hepatitis.	Moderate AST or ALT >2.5 times but ≤5 times ULN, or moderate total bilirubin elevation >1.5 times but ≤3 times ULN: withhold ipilimumab dose. Severe AST or ALT elevations of >5 times ULN; total bilirubin elevations of >3 times ULN; or failure to complete full treatment course within 16 weeks from administration of first dose: permanently discontinue ipilimumab. Grade ≥3 hepatitis: consider corticosteroid therapy.

Endocrine	Nonspecific symptoms include fatigue, headache, changes in mental status, abdominal pain, unusual bowel habits, and hypotension. Undertake appropriate blood work.	Moderate reactions or symptomatic endocrinopathy: withhold ipilimumab until complete resolution or stable on hormone replacement therapy. Patients unable to have their corticosteroid dose reduced to 7.5 mg prednisone or equivalent per day: permanently discontinue ipilimumab. Consider long-term hormone replacement therapy as necessary.

Neurologic	Encourage patient report of changes in muscle weakness or sensory alternations.	New onset or worsening symptoms: may require permanent discontinuation of ipilimumab.

Ocular	Assess patients for uveitis, iritis, or episcleritis.	Administer corticosteroid drops.

ALT: alanine aminotransferase; AST: aspartate aminotransferase; GI: gastrointestinal; LFTs: liver function tests; ULN: upper limit of normal.
